# Exceptional 20^th^ century glaciological regime of a major SE Greenland outlet glacier

**DOI:** 10.1038/s41598-017-13246-x

**Published:** 2017-10-19

**Authors:** Camilla S. Andresen, Ulla Kokfelt, Marie-Alexandrine Sicre, Mads Faurschou Knudsen, Laurence M. Dyke, Vincent Klein, Fanny Kaczmar, Martin W. Miles, David Wangner

**Affiliations:** 1 Geological Survey of Denmark and Greenland, Department of Glaciology and Climate, Øster Voldgade 10, 1350 Copenhagen K, Denmark; 2 Sorbonne Universités (UPMC, Univ. Paris 06)-CNRS-IRD-MNHN, LOCEAN Laboratory, 4 place Jussieu, F-75005 Paris, France; 3 Centre for Past Climate Studies, Department of Geoscience, Aarhus University, Høegh-Guldbergs Gade 2, 8000 Aarhus C, Denmark; 4 Uni Research Climate / Bjerknes Centre for Climate Research, Bergen, Norway; 5 Institute of Arctic and Alpine Research, University of Colorado, Boulder, CO USA

## Abstract

The early 2000s accelerated ice-mass loss from large outlet glaciers in W and SE Greenland has been linked to warming of the subpolar North Atlantic. To investigate the uniqueness of this event, we extend the record of glacier and ocean changes back 1700 years by analyzing a sediment core from Sermilik Fjord near Helheim Glacier in SE Greenland. We show that multidecadal to centennial increases in alkenone-inferred Atlantic Water SSTs on the shelf occurred at times of reduced solar activity during the Little Ice Age, when the subpolar gyre weakened and shifted westward promoted by atmospheric blocking events. Helheim Glacier responded to many of these episodes with increased calving, but despite earlier multidecadal warming episodes matching the 20^th^ century high SSTs in magnitude, the glacier behaved differently during the 20^th^ century. We suggest the presence of a floating ice tongue since at least 300 AD lasting until 1900 AD followed by elevated 20^th^ century glacier calving due to the loss of the tongue. We attribute this regime shift to 20^th^ century unprecedented low sea-ice occurrence in the East Greenland Current and conclude that properties of this current are important for the stability of the present ice tongues in NE Greenland.

## Introduction

In the last two decades there has been a rapid increase in the loss of ice from the Greenland Ice Sheet^1^. A large part of this change has been attributed to increased iceberg calving from tidewater outlet glaciers^2,3^, highlighting the role of marine-terminating glaciers in regulating ice-sheet stability. Mass change of the Greenland Ice-Sheet has only been continuously tracked with satellite data since the early 1990s. To understand whether the mass loss of the recent decade represents an outstanding event, or whether it is part of recurring phenomena acting on annual, decadal, or centennial timescales, we lack a longer-term history of the Greenland Ice Sheet. The answer to this question has potentially strong implications for the kind of ice-sheet changes we can expect in the context of future warming climate.

**Figure 1:**
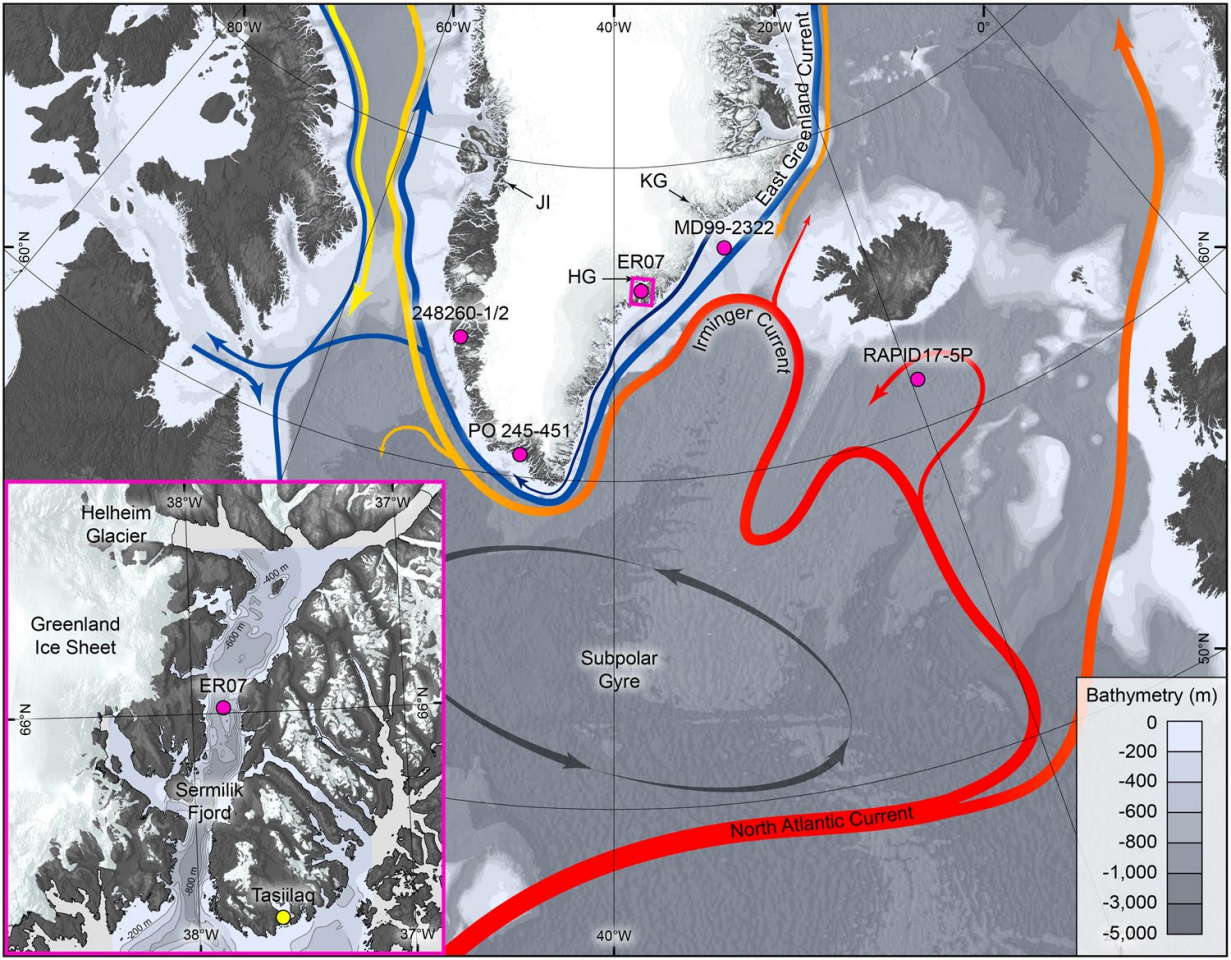
Map of the North Atlantic region showing the major surface ocean currents^13^ (colour of arrows indicate temperature; red = warm, blue = cold, yellow = mixture) and location of sites referred to in Fig. 3a–f. HG = Helheim Glacier; KG = Kangerdlugssuaq Glacier; JI = Jakobshavn Isbræ. Magenta circles show location of sediment cores discussed in the text and shown on Figs 2 and 3. The magenta box delineates the extent of the inset map. Bathymetric data are from IBCAO v3^56^. Terrestrial topographic data are from the ETOPO1 Global Relief model^57^ and the GIMP surface digital elevation model^58^. The inset map of Sermilik Fjord shows the location of sediment core ER07 and the local bathymetry^59^. The figure was created using *ArcMap 10*.1 (http://desktop.arcgis.com/en/arcmap/) and *Adobe Illustrator CS6 (* http://www.adobe.com/products/illustrator.html).

Concerns about glacier behavior emerged with the observation that many outlet glaciers on Greenland, including the three largest, suddenly increased their discharge at the turn of the 21^st^ century^4^. Specifically Jakobshavn Isbræ, in West Greenland, and Kangerdlugssuaq and Helheim glaciers in SE Greenland (Fig. 1), accelerated, thinned, and retreated between 1996 and 2005^1,4,5^. It was suggested that warming of subsurface ocean waters off West and SE Greenland triggered the acceleration and melting of these glaciers^6–8^. The North Atlantic subpolar gyre (Fig. 1) delivers warm and salty subtropical waters to SE and West Greenland via the Irminger Current (IC). Saltier waters by Greenland have been observed in the mid- 1990s^9,10^ and attributed to a westward movement of the subpolar frontal system, which was caused by a weakening of the subpolar gyre circulation^9,11^. As warm IC waters spread westward they penetrated further onto the SE and West Greenland continental shelf affecting the rate of submarine melting at glacier termini, thereby increasing iceberg calving rates and mass loss^12,13^.

The link between climatic changes and outlet glacier variability during the past century has been demonstrated by a reconstruction of Helheim Glacier calving based on the combination of three sediment cores from Sermilik Fjord^14^. This high-resolution record (up to 1 year) documents several episodes of increased iceberg calving of which the youngest marked episode occurred between 2000 and 2005. This study found that regional climatic modes (the North Atlantic Oscillation, NAO and the Atlantic Multidecadal Oscillation, AMO) may have influenced glacier calving through modulation of warm subsurface waters on the SE Greenland shelf and inside the fjord and that, conversely, episodes of increased cold polar waters occurrence may have potentially stabilised the glacier termini. However, almost nothing is known about the behaviour of Helheim Glacier before the 20^th^ century and thus how on-going climate changes will affect the dynamics of outlet glaciers.

Here we extend the 20^th^ century record of ocean and glacier variability back to 300 AD by analysing sediment core ER07 from Sermilik Fjord. Specifically, we use alkenone thermometry to reconstruct sea-surface temperature (SST). We assess the impacts of ocean conditions and sea-ice on the stability of Helheim Glacier using a reconstruction from the same core of iceberg sediment rafting while discussing the relationship between iceberg calving and sediment rafting. These new data provide the first-ever multi-centennial record of ocean and glacier changes that includes the 21^st^ century changes in detail and thereby places the current changes into a longer temporal context embedding low frequency ocean variability.

## Setting

Sermilik Fjord is situated in central SE Greenland; it is 80 km long, up to 15 km wide, and 600–900 m deep (Fig. 1). The two meter long sediment core ER07 was obtained from 525 m water depth (66°00′59″N, 37°51′07″W). Three outlet glaciers from the Greenland Ice Sheet drain into the north of the fjord; Helheim, Fenris, and Midgård Glaciers. Of these, Helheim Glacier is by far the largest and fastest and it is the third most profilic exporter of icebergs in Greenland^2^. The ocean waters that circulate on the SE Greenland shelf dominate the subsurface waters in Sermilik Fjord. The hydrological vertical structure of the fjord is characterised by a 10–20 m thick surface layer of glacial melt water, a 100–150 m thick intermediate layer of cold Polar water, and a lowermost layer of warm saline water derived from the IC^15^. The primary driver for SST variability in coastal SE Greenland is the varying influence of the IC and the East Greenland Current (EGC), the two major currents that dominate the local hydrology^14,16^.

### Variability in sea surface temperature in SE Greenland shelf waters during the past 1700 years

**Figure 2:**
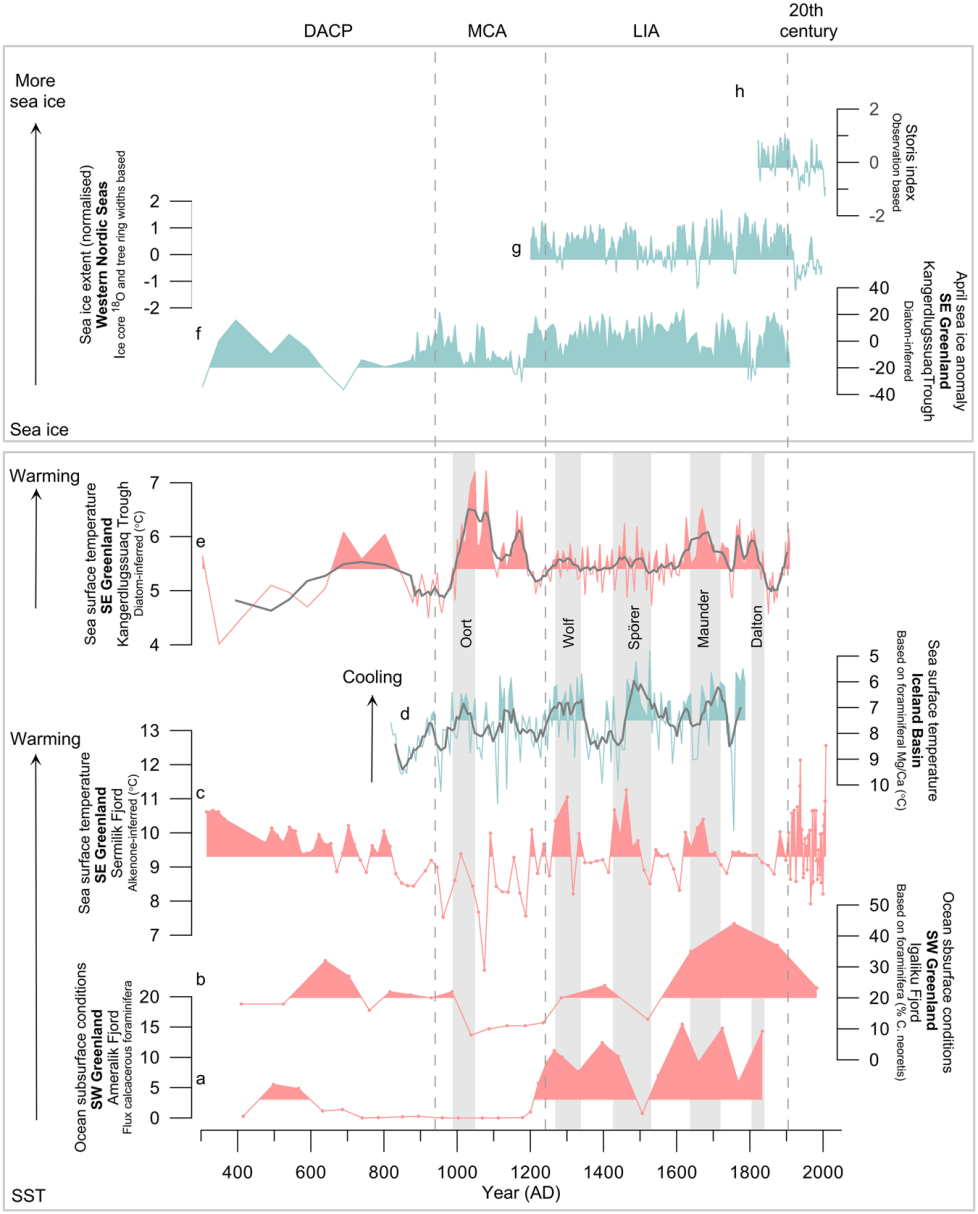
Paleoclimate records off and near Greenland. (**a**) Flux of calcareous foraminera in core 248260–2 (Ameralik Fjord, SW Greenland) as indicator of subsurface ocean warming^39^. (**b**) Relative amount (%) of foraminifera *C*. *neoteretis* in core PO 243–451 (Igaliku Fjord, SW Greenland) as indicator of ocean subsurface warming^40^. (**c**) Alkenone-based SST reconstruction from sediment core ER07 (Sermilik Ford, SE Greenland, this study). (**d**) Thermocline temperature reconstruction based in Mg/Ca in *G*. *inflata* from core RAPiD-17–5P plotted on a reversed Y axis (Iceland Basin)^28^. (**e**) Diatom-based August SST reconstruction from core MD99–2322 (Kangerdlugssuaq Trough, SE Greenland)^26^. (**f**) Diatom-based April sea-ice concentration anomalies in the same core as (**e**). (**g**) Greenland Sea winter sea-ice extent reconstruction of the Western Nordic Seas based on tree-ring widths and ice core ^18^O records from Fennoscandia and Svalbard, respectively^43^. (**h**) Fram Strait sea-ice export reconstruction from the Storis index; a proxy for sea-ice in the EGC^46^. Grey lines on (**d**) and (**e**) show 5-yr running means.

Alkenone biomarkers, mainly produced by the coccolitophorid *Emiliana huxleyi*, have successfully been used for SST reconstructions in this region^17,18^. This method was previously applied to core ER07 for reconstructing the SST variability of the last 100 years^17^. Our new 1700-year long record shows SSTs ranging from 6 to 12.6 °C (Fig. 2c), thus in a much larger range than the 0–4 °C measured today in the surface waters in Sermilik Fjord^16^. This difference, already noticed in the previous study^17^, has been explained by the advection of allochthonous alkenones, i.e. not produced in Sermilik Fjord. Indeed, these authors have shown that alkenones found in Sermilik Fjord sediments are sourced from the high-productivity oceanic frontal region of the continental shelf outside the fjord, where present-day SSTs range from 8 to 13 °C^19^ and advected into the fjord where they are deposited. Consequently, the alkenone-based temperature signal is indicative of the SST variability of the shelf waters entering the fjord^17^.

Our record shows multidecadal to centennial shifts in the temperature of surface ocean waters off SE Greenland with progressive cooling until the onset of the 20^th^ century (Fig. 2c). The time series has been divided into four time periods on the basis of the centennial scale changes. The first period, from 300 AD to 950 AD, is characterised by high SSTs (mean 9.6 °C), a long-term decrease and low variability (range 8.4–10.7 °C). This period corresponds in Northwest Europe to the so-called Dark Ages Cold Period (DACP). The subsequent interval, between 950 AD and 1250 AD, contains among the lowest SSTs in the record (mean 8.5 °C, range 6–10,1 °C) progressively increasing from 6 to 10 °C during the 11^th^ and 12^th^ centuries with a strong variability. This period broadly coincides with the so-called Medieval Climate Anomaly (MCA), or Medieval Warm Period (MWP)^20^ reported in numerous paleoclimate records though with different duration. Most MCA records, however, document warmer climatic conditions in the Northern Hemisphere, including near our study area in west and summit Greenland^21,22^ and off North Iceland^23^ with concomitant low sea-ice cover^24^.

The abrupt SST increase at the end of the 12^th^ century marks the transition to a new temperature regime from 1250 AD to 1900 AD. This interval coincides with the Little Ice Age (LIA), a period of general atmospheric cooling and glacier advance in most sites of the Northern Hemisphere and expansion of sea-ice in the North Atlantic Ocean^25^. The reconstructed SSTs during this interval are high (mean 9.4 °C; range 8.2–11.3 °C) and reveal a subtle cooling trend and greater variability than during the DACP. Three notable warm periods are salient around 1270–1300 AD, 1430–1460 AD, and 1620–1670 AD.

From the early 20^th^ century to present-day, average SSTs increased slightly (mean 9.8 °C, range 7.9–12.6 °C) to values that are comparable to the mean conditions during earlier warm episodes of both the DACP and the LIA. The higher variability of SSTs during the 20^th^ century could in part be due to the higher temporal resolution of the record in this interval (Fig. 2c).

Due to the scarcity of dateable material in glacioproximal sediments^14^C dating constraints before 1900 AD are sparse in core ER07 (Supplementary Table S1). To increase robustness in our age model in the interval 300–1900 AD we compared the Sermilik SST record with the more densely dated SST record from nearby Kangerdlugssuaq Trough core MD99–2322^26^, established from four ^14^C dates and one tephra layer (Fig. 1 and Fig. 2e). In similarity with the continental shelf offshore Sermilik Fjord, the Kangerdlugssuaq Trough warm waters originate from the Irminger Sea via the IC^13^. Therefore, it is expected that multidecadal ocean variability in the Irminger Sea and IC would be reflected in both SST records. The EGC is also expected to influence SSTs similarly at the two sites, although the generally higher sea-ice occurrence by the more northerly Kangerdlugssuaq Trough may explain differences in absolute SST values at the two sites. Comparison of the multidecadal SST variability in both records shows a highly significant positive correlation for the period after the MCA (1230–1900 AD) (R = 0.29; P = 0.03) when compared to randomly generated red-noise data, and a positive correlation for the period 300–900 AD (R = 0.42; P = 0.12), although only marginally significant, which is probably due to the low data-resolution in core MD99–2322 (see supplementary information for statistical analysis). These results strengthen our confidence in the age model for core ER07.

### Drivers of ocean variability off SE Greenland

**Figure 3:**
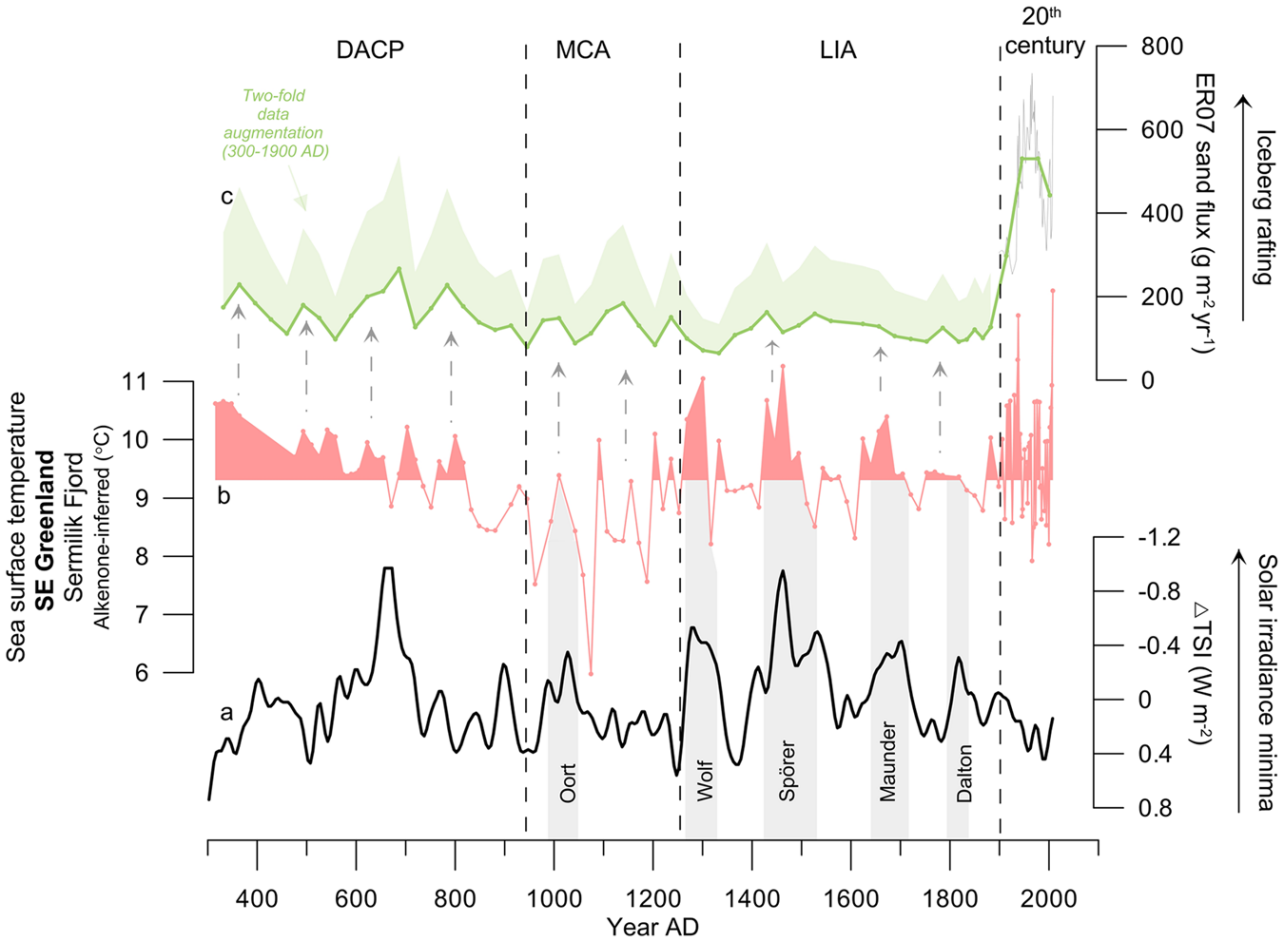
Core ER07 data from Sermilik Fjord and solar activity. (**a**) Solar irradiance reconstruction^60^. (**b**) SST reconstruction from core ER07 in Sermilik Fjord, SE Greenland based on alkenone thermometry (this study). (**c**) Green line: sand flux in core ER07 used as a proxy for relative iceberg rafting (green shading: reconstruction augmented 300–1900 AD). In order to compare the lower and higher resolution portions of the IRD record, a 32 years binning was applied in data younger than 1900 AD (32 year is the average time resolution of samples older than 1900 AD). The original raw data are shown as a thin grey line. Grey stippled arrows indicate concurrent ER07 SST and ice rafting peaks. Grey bars indicate periods when increased SST concurs with low solar activity.

The comparison of our new SST record with a reconstruction of past changes in total solar irradiance (TSI) (Fig. 3a,b) indicates that episodes of warmer SSTs occurred during periods of low solar activity. It is particularly striking that SST maxima are largely concurrent with the well-known Wolf, Spörer, Maunder and Dalton solar minima of the LIA. This visual match is underpinned by a highly significant negative correlation (R = −0.43; P = 0.02) between the TSI and our new SST record from Sermilik Fjord across the four solar minima that followed the MCA (1230–2000 AD). This relationship is further supported by wavelet analyses showing high common power in both records at periodicities between 120–210 years, and significant wavelet coherence at these periodicities, between 1230 and 1800 AD (Supplementary Figs S1 and 2).

Links between changes in solar activity and oceanic conditions around Greenland and the North Atlantic Ocean have previously been demonstrated^26–29^. The physical mechanisms at play remain debated^30^, but considering the small amplitude of the variations in TSI, amplifying mechanisms are likely to explain the magnitude of climate variability in paleo-records. Climate modelling has demonstrated that atmospheric circulation responds to low solar activity by resembling the negative phase of the winter NAO^31,32^. Other modeling studies have shown that solar minima of the 20^th^ century and the Maunder Minimum favor persistent North Atlantic anticyclonic blocking^33^. This weather regime has been related to the negative phase of the East Atlantic pattern (EA^-^) and shown to be responsible for the most severe winters on record in Europe^34^. This mode of variability has a similar North South dipole structure as the NAO but its centers of action are displaced southeastward. Both NAO and EA are important drivers of the North Atlantic Ocean circulation. The response of the ocean to weaker westerly winds (negative NAO) as well as persistent blocking events across the Atlantic is a wind-driven reduction and shrinking of the subpolar gyre^9,35,36^. Indeed, oceanic warming in West Greenland at the turn of the 21^st^ century has been paralleled to a decrease from high positive NAO index values in the early 1990s to negative state in 1996 and the strong decline of the subpolar gyre circulation^9^. In a recent study, the role of negative EA and blocked events on the subpolar gyre circulation has been evidenced^35^. Therefore, negative NAO and persistent blocking events favored by reduced solar activity are likely to explain the warm oscillations of the Atlantic waters seen off SE Greenland (Sermilik and Kangerdlugssuaq^29^) and the generally higher temperatures during the LIA than MCA off Sermilik Fjord. In contrast to SE Greenland, in the RAPID17–5P core south of Iceland, solar activity positively correlates with SSTs^28^ (Fig. 2d). This response to TSI changes is in accordance with a negative correlation between SST variations south of Iceland and offshore Sermilik Fjord (R = −0.26; P = 0.05) (see supplementary information). This finding underlines contrasting oceanic conditions between the SPG and the colder and fresher NAC^9^ as a result of the extension and shape of the SPG and subsequent position of the NAC, although more recent studies have shown that the eastern and western branch of the subpolar gyre may respond differently to atmospheric forcings^36,37^. Our record shows that both DACP and the LIA were characterised by high SSTs near Sermilik Fjord (Fig. 2c). Warming of sub-surface IC-sourced waters during the DACP and LIA has been reported at other sites on the Southern Greenland shelf ^38–40^ (Fig. 2a,b) suggesting that warmer and/or higher proportion of warm IC-sourced waters spilled onto the Greenland shelf. Indeed, temperature of the Greenland shelf is a function of both upstream NAC water temperature and the subpolar gyre strength. While high shelf SSTs during the LIA was linked with centennial scale weakening of the subpolar gyre induced by low solar activity, this may not have necessarily been the case during the DACP.

The intervening MCA period from 950 to 1250 AD of generally cold SSTs near SE Greenland (Fig. 2c) was characterized by a lesser influence of IC-sourced subsurface waters on the southern Greenland shelf ^38–40^ (Fig. 2a,b). This is in contrast with the generally warmer SSTs of the NAC due to increased northwards heat transport under stronger Westerlies that presumably prevailed during the MCA^41^. Therefore, cooling of the Greenland shelf waters indicates that stronger westerlies while expanding eastwards and strengthening the subpolar gyre would have diminished the inflow of IC-sourced waters onto the Greenland shelf. The generally lower sea-ice occurrence in the EGC during the MCA is reflected in the overall higher SST values, as for example, north of our core site, by Kangerdlugssuaq Trough^26^ (Figs 1 and 2e). SSTs at this location were modulated to a greater extent by sea-ice occurrence in the EGC with warm MCA being a period of broadly reduced sea-ice^26,42^ (Fig. 2f–h) as also observed off North Iceland^24^.

According to the new data from SE Greenland core ER07 presented here, the average 20^th^ century SSTs of IC-sourced waters off Greenland was high, but not any higher than during the earlier multidecadal episodes of warming. At the same time sea-ice occurrence in the EGC during most of the 20^th^ century was unprecedentedly low^43–46^ (Fig. 2g,h).

### 20^th^ and 21^st^ century mass loss from Helheim Glacier in a 1700 year context

To investigate the response of Helheim glacier over the past 1700 years of ocean changes near Sermilik Fjord the relative variability in glacier melt and iceberg production is assessed. This is done by reconstructing the relative variability in the amount of sediment-laden icebergs exciting the fjord. A high-resolution calculation of the sand flux (i.e. the amount of sand deposited per area sea-bed per year; Supplementary Fig. S3) in the sediment core (Fig. 3c) is generated, relying on the assumption that sand grains are too large to be advected in the meltwater plume, and consequently must have been rafted into the fjord by icebergs. This method was applied in a previous study combining three sediment cores from Sermilik fjord (including ER07)^14^ (Supplementary Figs S3 and S4) and demonstrated the applicability of ice rafted debris (IRD) in Sermilik Fjord as a proxy for iceberg production (calving) from Helheim Glacier.

The 1700-year long record from Sermilik Fjord reveals a decrease in iceberg rafting from 300 AD to 1300 AD (Fig. 3c). This is followed by a period of stabilized iceberg debris accumulation that terminates with a marked increase at around 1900 AD; this final phase persisted through the 20^th^ century to the present. A pronounced increase in sedimentation rate during the 20^th^ century is also found in a more southerly core from Sermilik Fjord (Supplementary Figs S4 and S5). Multidecadal variability prevails in the ER07 ice rafting signal and many of the relatively short-lived ice rafting episodes coincide with short-lived SST peaks (Fig. 3c). Near-synchronous pattern of calving in Sermilik Fjord, negative NAO index, and warming of the IC near Greenland was evidenced during the 20^th^ century^14^. Based on comparison to red-noise data, however, a statistically significant relationship between ice-rafting episodes and SST variations cannot be recognised for the entire period prior to the 20^th^ century (see supplementary information). We suggest that those of the pre-20^th^ century multidecadal ice rafting episodes that are concurrent with increased SSTs relate to climatically forced increased iceberg calving. This indicates that some ice rafting events may be related to intrinsic glacier behavior and that some of the IC warming episodes did not result in glaciological changes.

The cause of unprecedented levels of iceberg rafting throughout the 20^th^ century is unexpected since average SSTs during the 20^th^ century were similar to those during previous multidecadal warming episodes. We suggest that the unprecedented high iceberg rafting from Helheim Glacier after 1900 AD links with unprecedented reductions in sea-ice in the EGC after 1900 AD (Fig. 2g,h). The lowered sea-ice abundance in the EGC during the 20^th^ century may have increased air temperatures markedly in coastal areas of SE Greenland after 1900 AD, as supported by lake sediment studies nearby Sermilik Fjord designating the 20^th^ century warming as a conspicuous feature over several millenia^47,48^.

Thus the higher sea-ice abundance in the EGC and relatively low air temperatures in coastal areas of SE Greenland prior to the early 1900s, may have suppressed iceberg calving in addition to depriving icebergs of sediment; together resulting in low levels of iceberg rafting during this interval. Modern observations demonstrate that air cooling and attendant decrease in meltwater production can inhibit glacier discharge; this occurs through a reduction in hydrofracturing of crevasses^49^ and an increase in basal friction^50^. Lower air temperatures may also increase the rigidity of the mélange of icebergs in front of Helheim glacier^51^. This would serve to stabilise the glacier margin and decrease calving during cold climatic conditions. However, the relatively limited trimlines around the terminus of Helheim Glacier indicates that its mass loss since the LIA has been relatively low, especially when compared with the large post-LIA mass loss from Jakobshavn Isbræ and Kangerdlugssuaq Glacier^52^. This observation indicates that the nearly four-fold increase in iceberg rafting in Sermilik Fjord after the LIA may not be explained exclusively by increased iceberg calving. We therefore suggest that Helheim Glacier may have developed a floating ice tongue in response to increases in sea-ice and cold atmospheric conditions between 300 AD and the early 1900s. Under this scenario melt-out of debris-rich basal ice from the glacier margin would be expected to increase resulting in a reduction in iceberg sediment content. Finally, if cold coastal air temperatures prior to the early 1900s increased the cohesiveness of the glacial mélange and resulted in local enhanced sea-ice cover beyond the mélange, this would increase iceberg residence times in the upper-fjord. Increased residence time of icebergs may result in enhanced melt-out of debris before icebergs transit the core site and thereby decrease iceberg rafting.

The reconstruction of iceberg rafting from Helheim Glacier together with SSTs of the shelf waters allows us to set recent dramatic glacier changes in SE Greenland into a longer-term context. It was recently found that the 2000–2005 AD increase in glacier calving may have been matched in magnitude by a similar event in the 1930s^14^. However, the current study indicates that elevated levels of iceberg calving during the 20^th^ century were unprecedented in the last 1700 years. Moreover, our new record shows that the high 20^th^ century SSTs were matched during several previous multidecadal warm episodes during the DACP and LIA, yet the IRD record demonstrates that Helheim Glacier behaved differently in the 20^th^ century. We attribute this finding to the presence of a relatively marked floating tongue at Helheim Glacier at least since the DACP, lasting until the abrupt end of the LIA in the early 20^th^ century and suggest that the glaciological regime shift was triggered by unprecedented low sea-ice concentrations in the EGC during the 20^th^ century. Part of the mass loss before the 20^th^ century would have taken place via submarine melt underneath this floating tongue, similar to the mass loss from more northerly ice streams of the Greenland Ice Sheet, such as Nioghalvfjerdsbræ Glacier^53^. Our results imply that model predictions of dynamic loss from ice streams in North Greenland need to account for threshold effects from sea-ice to better predict the future evolution of Greenland coastal glaciers in a warming North Atlantic Ocean.

## Material and Methods

Sediment core ER07 was obtained using a Rumohr corer, using 80 mm core liner, from 525 m water depth (66°00′59″N, 37°51′07″W). X-ray imagery^14^ (Supplementary Fig. S6) shows that the core consists of massive diamicton facies with abundant pebbles. A very sandy unit occurs below an erosional boundary at 124 cm. The upper-124 cm represents the last 1700 years of sedimentation, whereas a single radiocarbon date indicates an age of the sediment below the erosional boundary of ~8800 cal. yrs BP (Supplementary Table S1). The chronology of core ER07 was constructed by combining a^210^Pb model of the upper 25.5 cm^14^ (Supplementary Table S2) with the radiocarbon date from level 120 cm in the core (Supplementary Fig. S6, Supplementary Table S1) using linear interpolation. The temporal resolution ranges from 1 to 4 years in the interval dated by^210^Pb, and on average 16 years in the lower portion of the core constrained by ^14^C dating. The associated sedimentation rate for the same periods (before and after AD 1900) is 0.06 cm yr^−1^ and 0.13–0.5 cm yr^−1^ (average 0.25 cm yr^−1^), respectively. Material for grain size analysis was continuously sampled at intervals of 0.5 cm down to 26 cm, at 1 cm intervals between 26 and 31 cm, and at 2 cm intervals below 31 cm. The grain size distribution (volume %) was estimated by a Malvern Mastersizer 2000 laser particle sizer. Samples were dispersed with natrium pyrophosphate (0.01 M Na_4_P_2_O_7_ × 10 H_2_O) and treated in an ultrasound bath for two minutes before measuring the 0.3–1000 µm fraction. The flux of sand (>63 µm) was calculated as a function of the wt % of sand, sedimentation rate and dry bulk density.

Alkenones comprise a suite of methyl and ethyl C_37_ to C_39_ ketones with two or three double bonds that are produced primarily by the ubiquitous marine coccolithophorides *Emilinia huxleyi*. SSTs were obtained from the unsaturation ratio of C_37_ alkenones (${{\rm{U}}}_{37}^{{\rm{K}}^{\prime} }$ = C_37:2_/(C_37:2_ + C_37:3_) and the global calibration (T = (${{\rm{U}}}_{37}^{{\rm{K}}^{\prime} }$ − 0.039)/0.034))^54^, a well-established temperature proxy in paleoceanographic studies. Precision based on triplicate injections on selected samples has been estimated to be 0.3 °C. The SST reconstruction was built from the downcore ${{\rm{U}}}_{37}^{{\rm{K}}^{\prime} }$ values acquired at a sample step of 1 cm. Biomarkers were extracted from 5 gr of freeze-dried sediments with a mixture of methanol/methylene chloride (1:2 v/v). Alkenones were isolated from the total lipid extract by open column silica gel chromatography using solvent mixtures of increasing polarity. Gas chromatography analyses were performed to achieve individual alkenone separation and subsequent quantification. Details on the analytical procedure can be found in ref.^55^. The recent 100 years of the grain size and alkenone data were published in refs.^14,17^.

Binning of IRD data younger than 1900 was done by calculating the average sand flux in the following time intervals 1898–1930, 1931–1962, 1963–1996, 1997–2008 (AD).

## Electronic supplementary material

Supplementary Information
