# Supplementary material for: Exceptional 20th century glaciological regime of a major SE Greenland outlet glacier
Source: Sci Rep. 2017 Oct 19;7:13626. doi: 10.1038/s41598-017-13246-x (PMC5648833; doi:10.1038/s41598-017-13246-x)
Supplement: Supplementary file 1 — Supplementary Information [file 41598_2017_13246_MOESM1_ESM.pdf]

Supplementary information

Exceptional 20th century glaciological regime of a major SE Greenland outlet glacier

Camilla S Andresen[1*+], Ulla Kokfelt[1], Marie-Alexandrine Sicre[2], Mads Faurschou Knudsen[3], Laurence M. Dyke[1], Vincent Klein[2], Fanny Kaczmar[2], Martin M. Miles[4] and David Wangner[1]

[1]*Geological Survey of Denmark and Greenland, Department of Glaciology and Climate, Øster Voldgade 10, 1350 Copenhagen K, Denmark*

[2]*Sorbonne Universités (UPMC, Univ. Paris 06)-CNRS-IRD-MNHN, LOCEAN Laboratory, 4 place Jussieu, F-75005 Paris, France.*

[3]*Centre for Past Climate Studies, Department of Geoscience, Aarhus University, Høegh-Guldbergs Gade 2, 8000 Aarhus C, Denmark*

[4]*(1) Uni Research Climate / Bjerknes Centre for Climate Research, Bergen, Norway. (2) Institute of Arctic and Alpine Research, University of Colorado, Boulder, CO, USA*

*csa@geus.dk

Correlation and statistical significance

The correlation between the new Sermilik Fjord SST record and SSTs from the Kangerdlugssuaq Trough is computed after interpolating both records onto a common time grid with a 10-year equidistant spacing, and subsequently detrending of the interpolated data with a 5th degree polynomial function. The significance of the resulting Pearson correlation coefficient is evaluated by comparing the correlation to that obtained for synthetic red-noise data following the procedure described in Knudsen et al. (2014). The synthetic data are modelled as a first-order autoregressive (AR1) process following the procedure of Schulz and Mudelsee (2002) with characteristic memory factors (ϕ) equal to those obtained for the interpolated SST data from Kangerdlugssuaq. The characteristic memory factors (ϕ) are obtained using the covariance function for the AR1 process given as

$$\mathrm{cov}(\, X(t_i), X(t_j)\,) = \Phi_0 \times \phi^{|j-i|}$$

where Φ_0 is the variance and cov(X(t_i), X(t_j)) is the covariance observed at |j - i| time steps from zero lag. A total of 1,000 synthetic SST data sets are simulated for the Kangerdlugssuaq SST data using a Monte Carlo approach to determine the innovation factor and the initial value. The correlation coefficients obtained between the Sermilik and the synthetic SST data are compared with those obtained for the real SST data from Kangerdlugssuaq. These correlations, which are computed for the entire record as well as for the following intervals 310-900 AD, 900-1230 AD, and 1230-1900 AD separately, are used to estimate the probability (P) of obtaining the correlations by chance. The distinction of these three intervals is done to account for the long-term trend in sea ice occurrence by Kangerdlugssuaq Trough resulting in generally lower absolute values in SSTs before and after the MCA at this location. The positive correlation between Sermilik and Kangerdlugssuaq SST records for the period after the MCA (1230-1900 AD) is the only correlation that is highly significant when compared to red-noise data (97%, P = 0.03). The correlation is also positive for the period 300-900 AD, although only marginally significant (R = 0.42; P = 0.12), while it is negative and insignificant for the brief period associated with the MCA. The change in correlation around the termination of the MCA roughly coincides with an increase in the temporal resolution of the Kangerdlugssuaq record, which may influence the correlations prior to and during the MCA and render them more uncertain. In summary, the positive significant correlation between Sermilik Fjord and Kangerdlugssuaq Trough multidecadal SST variability over

the intervals DACP and LIA represents 86% of the investigated time interval (1370 years out of 1600 years), which reinforces our confidence in the age model of core ER07.

Care should be taken in interpretation of the statistically insignificant negative correlation between Sermilik and Kangerdlugssuaq multidecadal SST variability during the MCA. Yet we speculate that it may reflect a change in the hydrological conditions between Sermilik and Kangerdlugssuaq due to lower sea ice cover in the northern Kangerdlugssuaq Through as a result of the northern shift of the polar front at this time, also seen off North of Iceland (Sicre et al. 2014). We note, however, that Miettinen et al. (2015) link an exceptionally high SST peak around 1040 AD by Kangerdlugssuaq Trough with the Oort Solar Minimum (1000-1050 AD). Similarly, off Sermilik the generally cold MCA SST displays a marked shortlived SST maximum synchronously with the Oort Minimum. If these two SST peaks are the same event and thus positively correlated, this indicates that the statistic anticorrelation only applies to the later interval of the MCA around 1080-1250 AD; a time interval that concurs with the 'Medieval Solar Maximum' at 1100-1250 AD (Jirikowic and Damon, 1994). These findings of MCA climate events in both sediment cores may add further to the confidence in the ER07 agemodel.

The correlation and associated significance tests described in the main text are all computed according to the procedure described above. The correlation between the Sermilik SST record and changes in TSI variations are very significant (98%, P= 0.02) for the time interval following the MCA (1230-2000 AD), whereas no significant correlations between Sermilik SSTs and TSI variations can be found in the period before or during the MCA, suggesting a lesser role of TSI on SST variability. The correlation between the Sermilik SST and IRD records is positive and relatively high for data in the time interval 300-1900 AD that are not detrended, but the correlation does not emerge as significant when compared to red-noise data (82%, P = 0.18). The correlation between the Sermilik SST record and the SST record from the RAPID17-5P core south of Iceland for the period 820-1780 AD reveals a significant negative correlation (95%, P = 0.05) when the two records are not detrended but interpolated onto a common 10-year equidistant timescale. If the two SST records are detrended with a 5th degree polynomial function, the correlation remains negative, but comparison to red-noise data indicates that the multidecadal-scale correlations are only marginally significant (P=0.15).

Wavelet analyses

Wavelet analyses of the Sermilik and Kangerdlugssuaq SST records are carried out by applying the cross-wavelet and wavelet coherence software package of Grinsted et al. (2004). In these analyses, we use data from the interval 1230-2000 AD that have been detrended and interpolated onto a common 10-year equidistant timescale. Following standard procedures, a Morlet wavelet (a Gaussian-modulated sinewave) with a non-dimensional frequency of 6 ($\omega_0 = 6$) is used as the mother wavelet. The time series are padded with zeros to dampen edge effects and spectral leakage, which results in underestimation of the lowest frequencies near the edges. The cone of influence defines the region that is likely to be influenced by this effect. The cross-wavelet results show high common power at periodicities between 120 and 220 years in the time interval 1230-1820 AD that is highly significant (>95%) when compared to red-noise AR1 data. Similarly, the wavelet coherency analysis indicates significant (>95%) coherence between Sermilik SSTs and TSI variations at roughly the same periodicities in the interval 1230-1750 AD. This highly significant common power and wavelet coherence are partly found within the cone of influence, where edge effects may influence the wavelet power. However, as the records have been padded with zeros, the analyses are much more likely to underestimate the power of the periodicities between 120 and 220 years than to introduce them as spurious edge effects. Also, the fact that the significant periodicities are similar inside and outside the cone of influence, and that they occur at the same time in both analyses, strongly suggest that they reflect real periodic behavior in the data and not edge effects.

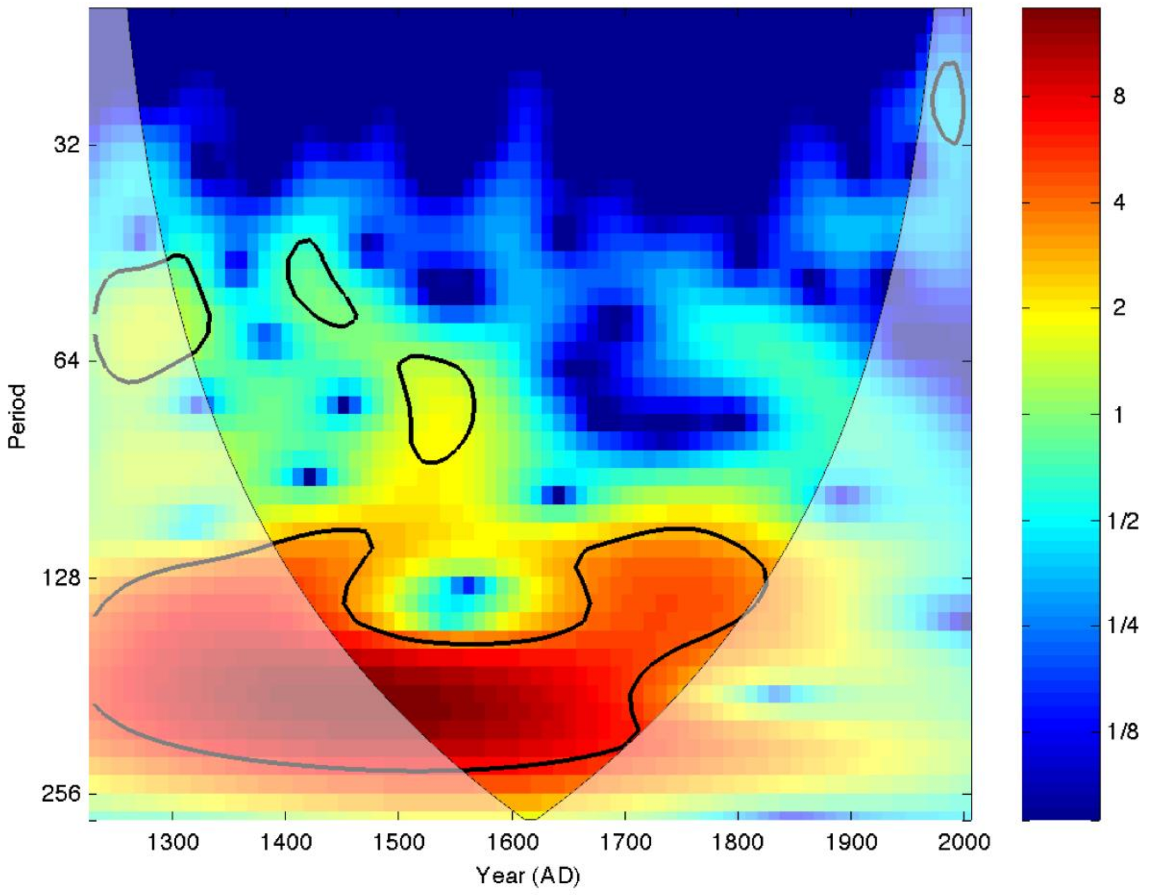

Supplementary Fig. S1. Cross-wavelet analysis of the Sermilik and Kangerdlugssuaq SST records.

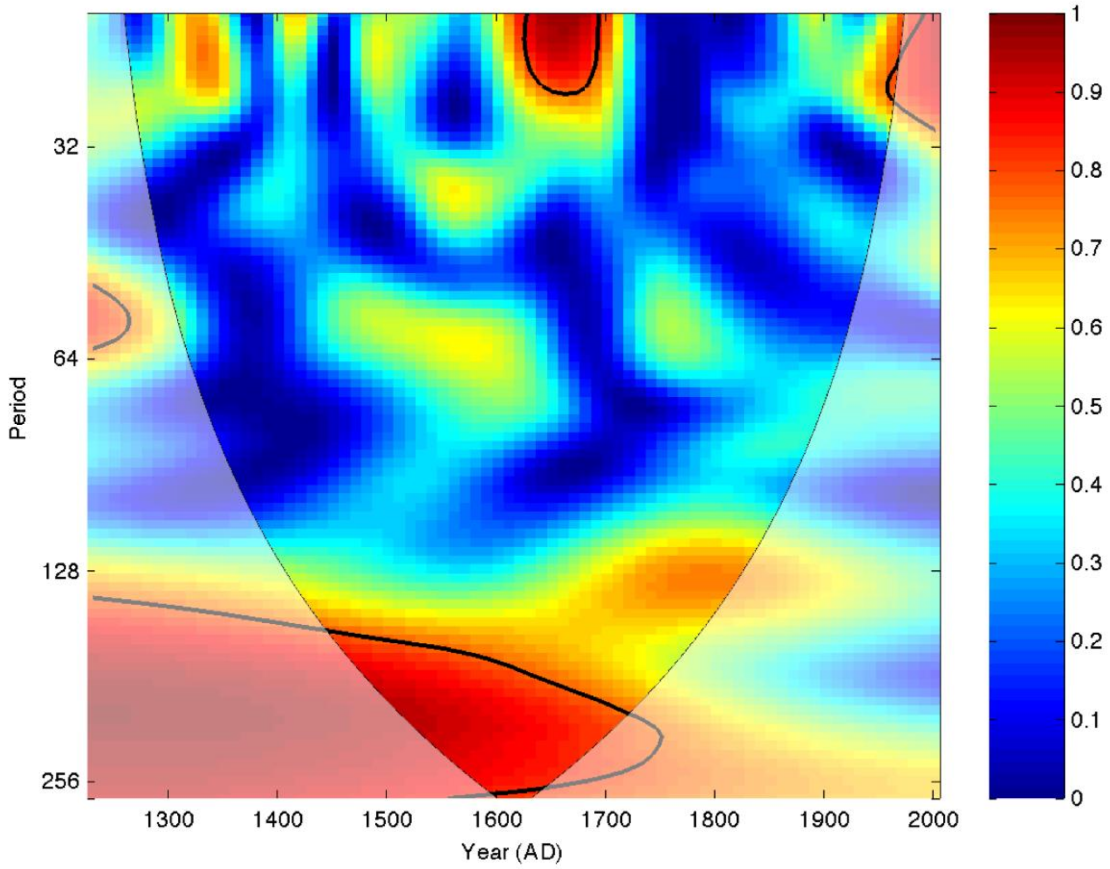

Supplementary Fig. S2. Wavelet-coherence analysis of the Sermilik and Kangerdlugssuaq SST records.

20th century iceberg calving as a function of ice rafting

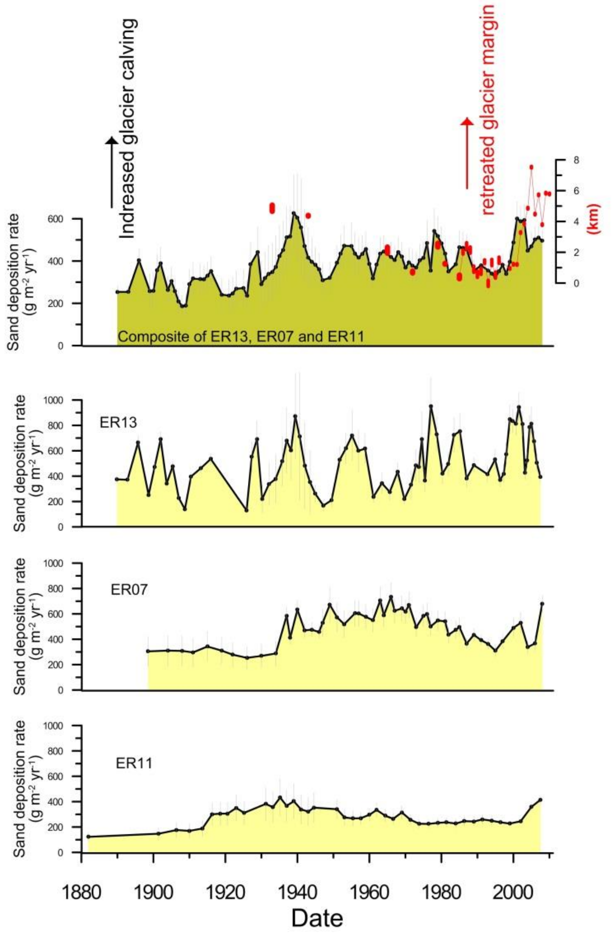

Supplementary Fig. S3. Reconstructed calving record of Helheim Glacier calculated as the average flux in the three cores (Andresen *et al.,* 2012, Supplementary Fig. S4). The flux is estimated as the amount of sand (63–1,000 micron) deposited per area sea-bed per yr (g m^{-2} yr^{-1}). This provides a better estimate for material deposited from icebergs than bulk sedimentation rate (thickness of sediment layer deposited per year), since the thickness of the sediment layer is additionally a function of deposition of clastic material via other processes than iceberg rafting (f.x. melt water plume sedimentation), as well as water content and compaction. Error bars are a function of 1 sigma error of mass accumulation rates and sand content. The glacier margin positions (red) are relative to the 1993 position according to aerial and satellite images. Comparison of the iceberg rafted debris (IRD) record with satellite images of the Helheim Glacier margin shows a correlation between glacier retreat and increased IRD deposition, which indicates the applicability of IRD as a proxy for glacier calving between 1890 to 2008 (Andresen *et al.,* 2012).

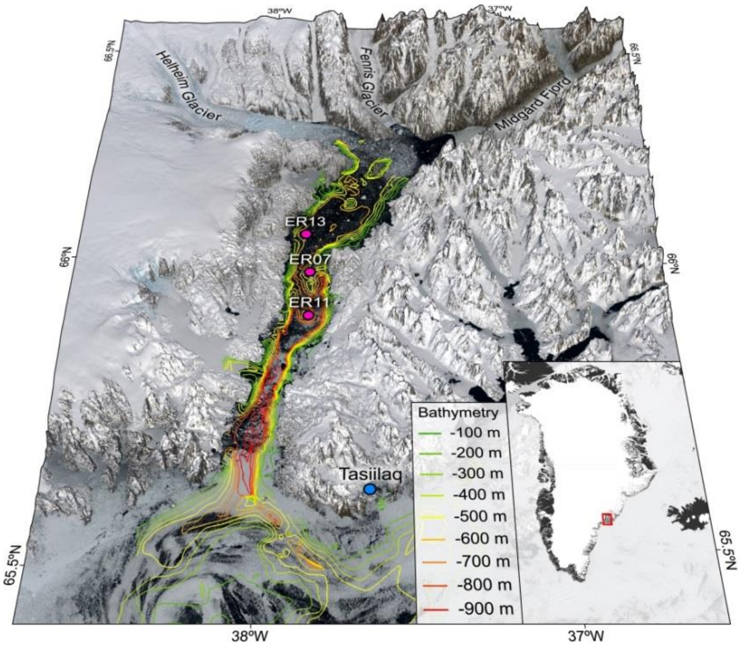

Supplementary Fig. S4. Supplementary Fig. S4. Map of Sermilik Fjord showing the location of sediment cores ER13, ER07 and ER11 (Andresen et al., 2012). Bathymetric data are from Schjøth et al. (2012). Landsat 8 imagery (NASA Landsat Program, 2016, Landsat 8 OLI scenes, LC82320132016104LGN00 and LC82320142016104LGN00, Level 1G, USGS, Sioux Falls, 13/04/2016) was projected onto the GIMP DEM (Howat et al., 2014) using ArcScene 10.1 (http://www.esri.com/software/arcgis/extensions/3danalyst). The figure was finalised in Adobe Illustrator CS6 *(http://www.adobe.com/products/illustrator.html).*

Estimating sedimentation rates in core ER11

Core ER11, obtained ~ 10 km south of core ER07 in the fjord (600 m water depth, 65°55'29''N, 37°50'52''W, Supplementary Fig. S4) is used for comparison to evaluate if the marked increase in sedimentation rate in the 20th century recorded in core ER07 is also seen at other locations of the fjord.

The upper part of core ER11 and ER07 have been dated using ^{210}Pb (Andresen et al., 2012, Supplementary Table S2) providing an absolute age constraint of c. 1900 AD at 22 cm and 25 cm depth, respectively (Supplementary Fig. S5a). Core ER07 was furthermore radiocarbon dated at 120 cm (See Supplementary Tables S1 and S2 and method section for construction of age model). ER11 could not be radiocarbon dated below depth 22 cm due to sparsity of dateable material. Therefore, we compared the XRF signals of ER07 and ER11 in order to evaluate the sedimentation rates of ER11. When XRF pattern are similar in the two cores the ages of core ER07 is applied to core ER11 and the sedimentation rate estimated for core ER11.

The split core halves were scanned at 1 cm resolution on an AVAATECH x-ray fluorescence (XRF) core scanner using the 10eV settings at the Royal Institute for Sea Research (NIOZ) in Texel, The Netherlands. This method provides an estimate of the chemical composition of the sediment in terms of element intensities in counts per second (cps) (Richter *et al.*, 2006). In order to avoid bias on XRF data from water content, elemental ratios were used for XRF comparison between the cores. Si/Al and Si/K and K/Ti ratios often fluctuate in parallel with grain size; Si tends to reside as SiO2 phases in the sand fraction, while Ti bearing phases (titanomagnetite, Ti-oxides, heavy minerals) tend to be associated with the silt fraction and K-bearing mineral phases most often to the clay fraction as clay minerals (Richter et al., 2006).

Comparison of XRF ratios along ER07 and ER11 cores reveal similarity (Supplementary Fig. S5a). The x-rays are plotted on Supplementary Fig. S5a in order to understand if marked peaks are caused by overall change in the grain size or if a local rock is influent. In the latter case, the event is reported on the figure. The correlation is based on the overall similarity of the XRF pattern throughout the entire length of the two cores. This means that correlation is based on comparing the size of the peaks as well as the succession and general peak pattern.

The sedimentation rate of core ER11 is estimated using the ages of ER07 at the levels featured by red stipled lines (Supplementary Fig. S5b). This approach indicates that the marked 20th century increase in sedimentation rate is not a unique feature in core ER07, and strengthens the argument that the increase in ice rafting in ER07 is related to changes in the glacier margin.

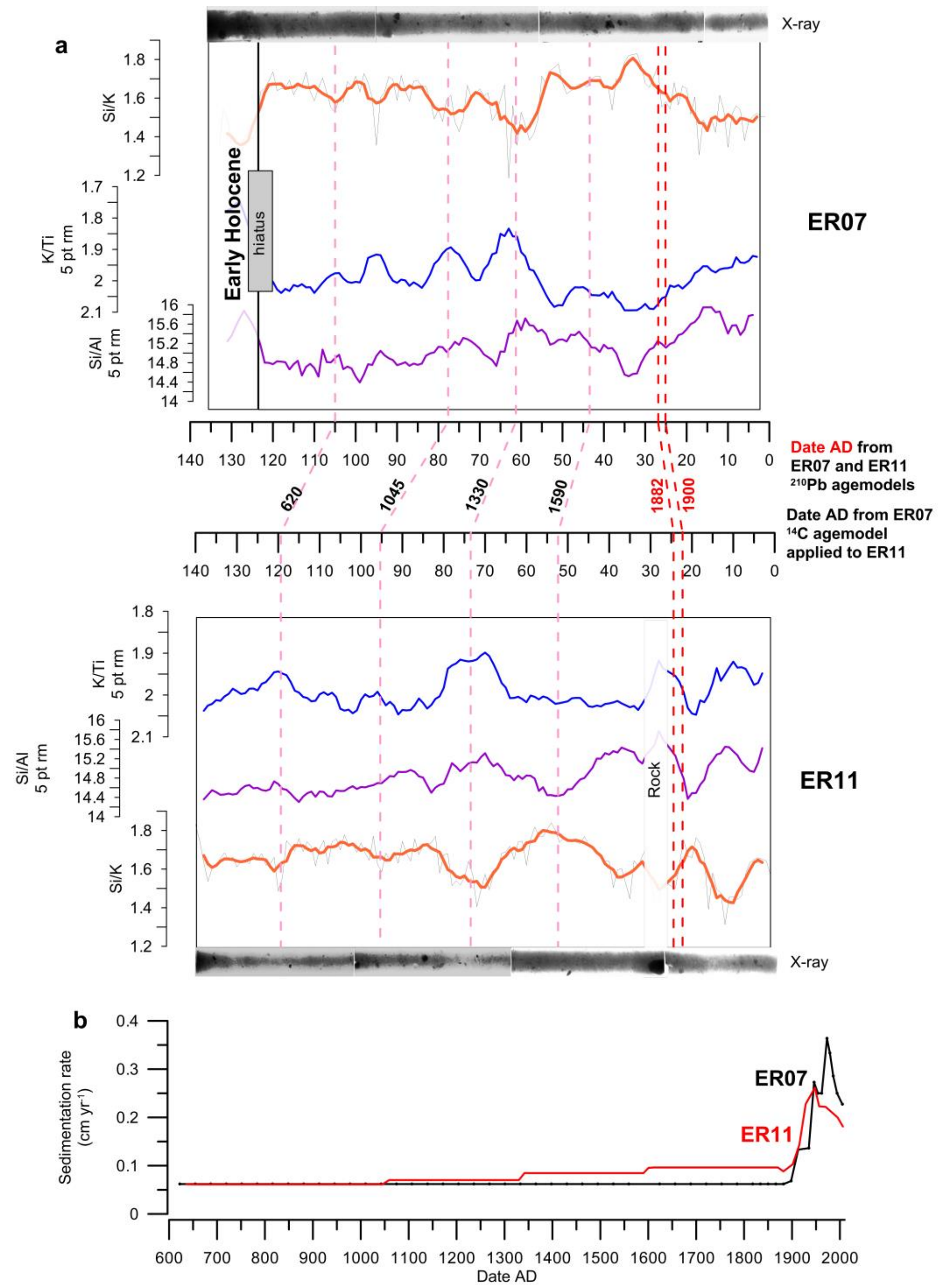

Supplementary Fig. S5. ER07 and ER11 data for comparison. (**a**) XRF ratios (5 pt running means) and x-rays. (**b**) Sedimentation rates for both cores. For the 20th century, sedimentation rates are calculated on the basis of ^{210}Pb dated levels in each core (Supplementary Table S2). Prior to the 20th century, sedimentation was calculated from the ^{14}C age for ER07; ER11 sedimentation rates are calculated between tie points resulting from the cross-analysis of ER07 and ER11 XRF data (red stipled lines).

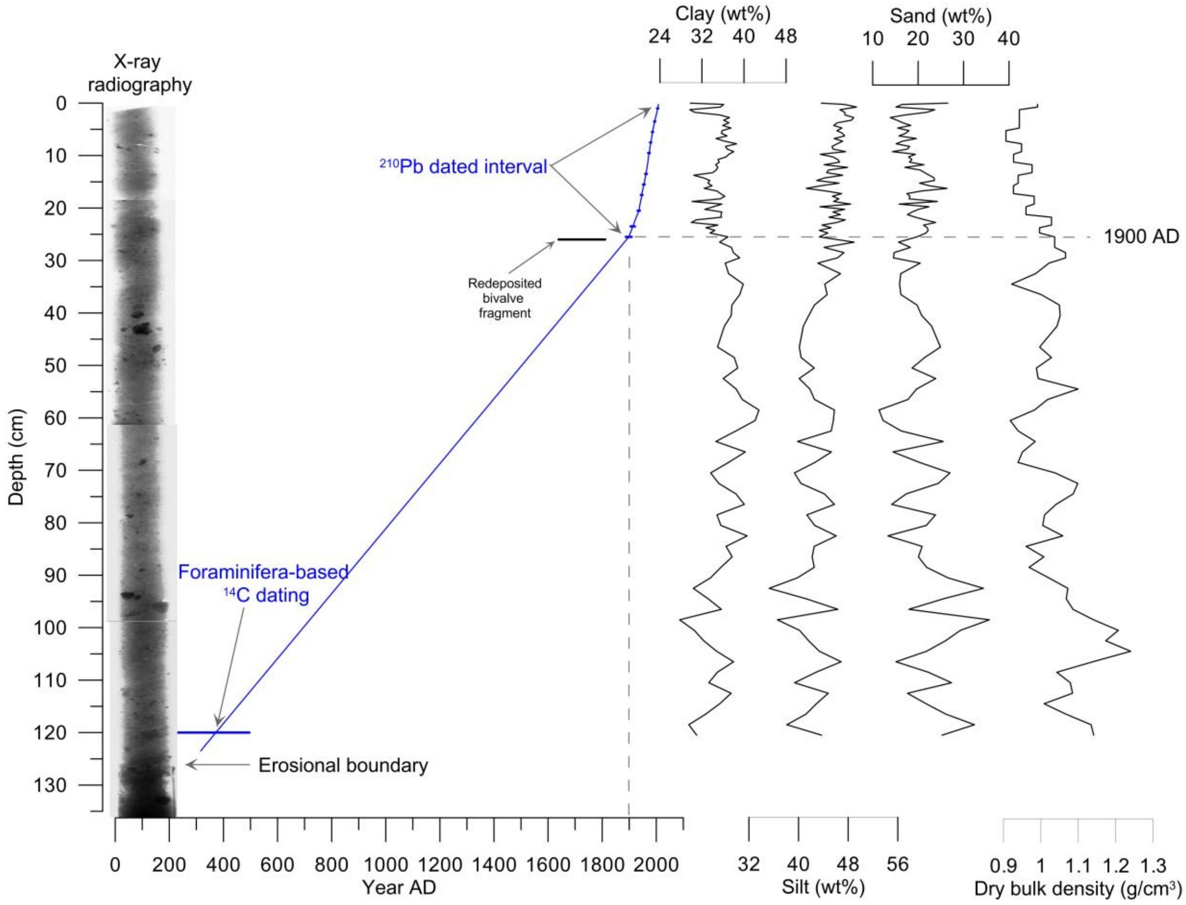

Supplementary Fig. S6. ER07 sediment data. X-ray, age model, wt% silt, clay and sand and dry bulk density. The age model (blue line) was constructed by combining the ^{210}Pb model of the upper 25.5 cm with the radiocarbon date from level 120 cm using linear interpolation. The marine bivalve fragment date at 26 cm (Table S1) is interpreted as being redeposited since it is older than the more robust ^{210}Pb date from 25.5 cm (Andresen *et al.* 2012) (Table S2).

Supplementary Table S1. Radiocarbon dating ER07

Lab ID	Material dated	Depth (cm)	^{14}C age (yrs BP)	error ±	2σ Calibrated* age interval (yrs BP)
Beta-431626	Marine bivalve fragment	26	610	30	139 - 311
AAR-13848	Mainly benthic forams	120	2000	120	1696 - 1411
AAR-13556	Mainly benthic forams	132	8225	46	8843 - 8640

*Marine calibration curve from Reimer *et al.*, (2004) using a reservoir correction of 400 yr (Jennings *et al.* 2006, Jennings *et al.* 2011). Radiocarbon dating was conducted at the AMS ^{14}C Dating Centre, Aarhus University, Denmark and at the Beta Analytical Radiocarbon Dating Laboratory, Miami, Florida.

Supplementary Table S2. ^{210}Pb dating (Andresen *et al.*, 2012)

^{210}Pb dating ER07

Depth (cm)	Age (year)	Error age (year)	Date
0			2009
1	4	2	2005
3.5	15	2	1994
5.5	23	2	1986
7.5	30	2	1979
9.5	36	3	1973
13.5	47	3	1962
15.5	55	3	1954
17.5	63	3	1946
20.5	74	4	1935
23.5	96	8	1913
25.5	111	11	1898

^{210}Pb dating ER11

Depth (cm)	Age (year)	Error age (year)	Date
0			2009
0.5	3	2	2006
2.5	14	2	1995
7.5	39	4	1970
10.5	52	4	1957
12.5	61	4	1948
17.5	80	6	1929
20.5	94	7	1915
22.5	108	7	1901
24.5	127	9	1882

References Supplementary Information

Andresen, C. S. *et al*. Rapid response of Helheim Glacier in Greenland to climate variability over the past century. *Nature Geoscience* **5**, 37-41 (2012).

Grinsted A., Moore J.C. & Jevrejeva S. Application of the cross wavelet transform and wavelet coherence to geophysical time series. *Non-linear Processes in Geophysics* **11**, 561–566 (2004).

Howat, I. M., Negrete, A. & Smith, B. E. The Greenland Ice Mapping Project (GIMP) land classification and surface elevation datasets. *The Cryosphere* **8**, 1509–1518 (2014).

Miettinen, A., Divine, D.V., Husum, K., Koç, N. & Jennings, A. Exceptional ocean surface conditions on the SE Greenland shelf during the Medieval Climate Anomaly. *Paleoceanography* **30**, 1-17 (2015).

Jirikowic, J.L., Damon, P.E.. The Medieval Solar Activity Maximum. *Clim. Change* **26**, 309 (1994).

Jennings, A. E., Hald, M., Smith, M. & Andrews, J. T. Freshwater forcing from the Greenland Ice Sheet during the Younger Dryas: evidence from southeastern Greenland shelf cores. *Quaternary Science Reviews* **25**, 282–298 (2006).

Jennings, A., Andrews, J. & Wilson, L. Holocene environmental evolution of the SE Greenland Shelf North and South of the Denmark Strait: Irminger and East Greenland current interactions. *Quaternary Science Reviews* **30**, 980–998 (2011).

Knudsen, M.F. et al. Evidence for external forcing of the Atlantic Multidecadal Oscillation since termination of the Little Ice Age. *Nature Communications* **5**, 3323 (2014).

Reimer et al. IntCal09 and Marine09 radiocarbon calibration curves, 0-50,000 years cal BP. *Radiocarbon* **46(3)**, 1029-1058. (2004).

Richter et al., 2006. The Avaatech Core Scanner: Technical description and applications to NE Atlantic sediments. In: Rothwell, R.G. (Ed.), New ways of looking at sediment core and core data. Geological Society Special Publication, London, pp. 39-50.

Schjøth, F. *et al*. Campaign to Map the Bathymetry of a Major Greenland Fjord. *EOS, Transactions American Geophysical Union* **93**, 141–142 (2012).

Schulz, M. & Mudelsee, M. REDFIT: estimating red-noise spectra directly from unevenly spaced paleoclimatic time series. *Computers and Geosciences* **28**, 421–426 (2002).

Sicre, M-A. *et al*. Labrador current variability over the last 2000 years. *Earth and Planetary Science Letters* **400**, 26-32 (2014).
